# Construction of Type-II Heterojunctions in Crystalline Carbon Nitride for Efficient Photocatalytic H_2_ Evolution

**DOI:** 10.3390/nano13162300

**Published:** 2023-08-10

**Authors:** Jingyu Zhang, Zhongliang Li, Jialong Li, Yalin He, Haojie Tong, Shuang Li, Zhanli Chai, Kun Lan

**Affiliations:** Inner Mongolia Key Laboratory of Chemistry and Physics of Rare Earth Materials, College of Chemistry and Chemical Engineering, College of Energy Materials and Chemistry, Inner Mongolia University, Hohhot 010021, China

**Keywords:** carbon nitride, heterojunction, build-in electric field, photocatalysis

## Abstract

As an encouraging photocatalyst, crystalline carbon nitride (CCN) exhibits unsatisfactory photocatalytic activity and stability due to its rapid recombination of photo-generative carriers. Herein, high-crystalline g-C_3_N_4_ was prepared, including CCN obtained in KCl (K-CCN), LiCl-KCl mixture (Li/K-CCN), and LiCl-NaCl-KCl mixture (Li/Na/K-CCN), via the molten salt strategy using pre-prepared bulk carbon nitride (BCN) as a precursor. The obtained BCN sample was formed by heptazine-based units, which convert into triazine-based units for K-CCN. Heptazine and triazine are two isotypes that co-exist in the Li/K-CCN and Li/Na/K-CCN samples. Compared with BCN and other CCN samples, the as-prepared Li/Na/K-CCN sample exhibited the optimal photocatalytic hydrogen evolution rates (3.38 mmol·g^−1^·h^−1^ under simulated sunlight and 2.25 mmol·g^−1^·h^−1^ under visible light) and the highest apparent quantum yield (10.97%). The improved photocatalytic performance of the Li/Na/K-CCN sample is mainly attributed to the construction of type-II heterojunction and the institution of the built-in electric field between triazine-based CCN and heptazine-based BCN. This work provides a new strategy for the structural optimization and heterostructure construction of crystalline carbon nitride photocatalysts.

## 1. Introduction

Photocatalytic hydrogen (H_2_) evolution from water splitting is regarded as an ideal strategy for future energy consumption [1,2,3]. As a traditional metal-free photocatalyst, polymeric graphitic carbon nitride (g-C_3_N_4_) has obtained more and more interest because of its high chemical and thermal stability, facile synthesis and modifications, visible-light response, and benign environmental behaviors [4,5,6,7,8]. Nevertheless, the photocatalytic applications of pristine g-C_3_N_4_ are greatly limited by the rapid electron–hole recombination rate and sluggish surface reaction kinetics due to less active surface sites [9,10,11]. Various approaches, such as doping with elements or introducing vacancies [12,13,14], designing heterojunctions with other semiconductors [15,16,17], and increasing the crystallinity [18,19], have been explored to improve the photocatalytic efficiency of g-C_3_N_4_. Unfortunately, its catalytic performance under visible-light irradiation still cannot meet the requirements for applications. One of the major reasons for this is the conflicting effects of crystallinity on the improvement in photocatalytic performance. Polymeric carbon nitride (PCN) prepared by typical polymerization or doping modification has good visible-light adsorption and a tunable electronic structure, but low crystallinity and high-density defects serve as recombination centers for photo-generated carriers, resulting in low charge transfer during photocatalytic water splitting [16,20]. Alternatively, high crystallinity carbon nitride (CCN) can effectively decrease the recombination center for radiative carriers, expand the light-harvesting range, and enhance the charge conductivity [21]. Although many modifications have been made in CCN [22,23], the obstruction of charge migration and separation still limits further performance improvement.

Essentially, PCN and CCN exhibit strong intrinsic Coulomb interactions, which are attributed to the shielding property and large dielectric constant [24]. Compared with the thermal interference energy of 25 meV at room temperature, the exciton binding energy of PCN and CCN is more significant at 100–500 meV, indicating the presence of photo-generated carriers as Frankel excitons, which dramatically impede the dissociation of photo-generated carriers in photocatalytic reactions [25]. Establishing built-in electric fields (BIEFs) by constructing heterojunctions is an effective strategy to improve the migration and separation of photo-induced carriers [26,27]. In fact, the construction of heterojunctions requires the matching of the energy level and interfacial atomic contacts [28,29], which makes it challenge to construct reasonable heterojunctions in g-C_3_N_4_. To the best of our knowledge, there are multiple subunit types in PCN, which have different energy-level structures and can modulate photocatalytic activity [30]. In general, the heptazine-based PCN with an extended π-conjugated system would be favorable for the light-harvesting and carrier migration/separation, compared to triazine-based PCN [31,32]. Nevertheless, the polymeric CCN obtained by the molten salt strategy was stabilized by the salt residues into a triazine-based structure [22]. Therefore, it is urgently desired to obtain a single heptazine-based CCN or a mixture of heptazine-based and triazine-based CCNs to improve photocatalytic performance. Recently, Wang et al. used pre-heated melamine as a precursor and molten salts as a high-temperature medium to prepare a heptazine-based CCN, which presented high visible-light photocatalytic activity toward H_2_ production [33]. Zhang et al. designed high-crystalline g-C_3_N_4_ by the molten salt approach using cyanuric acid-melamine as a precursor and introduced a built-in electric field in heptazine/triazine-based g-C_3_N_4_ [30]. Until now, there has been limited research on the preparation of heterojunctions in g-C_3_N_4_ using the molten salt method; as a result, the roles of the salts in the construction of heterojunctions are not entirely clear.

Herein, the molten salt strategy was explored for the crystallinity of CCN and the construction of heterojunctions between a heptazine-based PCN and a triazine-based CCN. Moreover, various types of molten salts (KCl, LiCl-KCl, and Li-NaCl-KCl) were adopted to investigate their effects on the catalyst structure and photocatalytic H_2_ production performance. Under simulated sunlight and visible-light irradiation, the activity and regeneration of the synthesized high-crystalline Li/Na/K-CCN were superior to those of other samples in photocatalytic H_2_ evolution. The optimal photocatalytic performances of Li/Na/K-CCN were explicated by the formation of a BIEF in the heptazine PCN/triazine CCN heterojunction for the improved separation of radiative carriers.

## 2. Experiments

### 2.1. Materials

Melamine (A. R.), lithium chloride (LiCl, A. R.), sodium chloride (NaCl, A. R.), and potassium chloride (KCl, A. R.) were purchased from Beijing InnoChem Science & Technology Co., Ltd. (Beijing, China). Chloroplatinic acid (H_2_PtCl_6_) was purchased from Beijing Inokai Technology Co. (Beijing, China). Triethanolamine (TEOA) was purchased from Tianjin Huihang Chemical Co., Ltd. (Tianjin, China). All chemicals are analytically pure without further purification.

### 2.2. Synthesis of Crystalline C_3_N_4_ (CCN)

A quantity of 5.0 g of melamine was loaded into a covered ceramic crucible and calcined for 2 h in a muffle furnace at 500 °C at a heating rate of 2 °C/min. The as-prepared sample was denoted BCN. A quantity of 2.0 g of the as-prepared BCN powder was ground with 6.6 g of anhydrous KCl or 13.2 g of a mixture of two salts (LiCl:KCl = 1:1) or 20.0 g of a mixture of three salts (LiCl:NaCl:KCl = 1:1:1) in an agate mortar. The prepared mixture was transferred into a 30 mL ceramic crucible and placed in a muffle furnace. The temperature was raised to 550 °C at a rate of 5 °C/min and held for 2 h. The prepared product was washed with hot water (80 °C) five times to remove the residual ions and dried in an oven at 60 °C. The synthesized products were denoted Li-CCN, Li/K-CCN, and Li-Na/K-CCN, respectively.

### 2.3. Material Characterizations

The microscopic morphologies were characterized by transmission electron microscopy (TEM, FEI Tecnai F20 S-Twin, 200 kV, Hillsboro, OR, USA). Zeta potentials were explored using a zeta potential and particle size analyzer (NanoBrook 90plus PALS, Bruker, Billerica, MA, USA). The crystal structures were determined by an X-ray diffractometer (XRD, PANalytical Empyrean, Malvern, UK) with a Cu K_α_ source, and the chemical valence states were tested by X-ray photoelectron spectroscopy (XPS, EscaLab 250Xi, PerkinElmer, Waltham, MA, USA) with Al K_α_ as the excitation source. The molecular structures were identified using Fourier transform infrared (FT-IR) spectroscopy (Bruker VERTEX 70V, Bruker, Billerica, MA, USA) at room temperature. Ultraviolet-visible spectroscopy (UV-vis) was undertaken with a spectrophotometer (Analytik Jena (Jena, Germany). Specord 50) within 200–800 nm, and the photoluminescence (PL) spectra were detected with a luminescence spectrometer (PerkinElmer LS 55, PerkinElmer, Waltham, MA, USA). The radical trapping at room temperature was recorded by a spectrometer (Bruker ER 200-SRC) irradiated with 420 nm. The electrochemical measurements were carried out on an electrochemical workstation (AutoLab, PGSTAT 302N, Metrohm, Herisau, Switzerland). Using Ag|AgCl as the reference electrode, Pt as the counterelectrode, FTO glass coated with 1 cm^2^ catalyst solution as the working electrode, and a 300 W Xenon lamp as a light source, the transient photocurrent responses and EIS Nyquist plots were determined. The photocatalytic properties were measured by an automatic photolysis system (Beijing Zhongjiao Jinyuan (Beijing, China), CEL-SPH2N).

### 2.4. Photocatalytic Measurements

The photocatalytic activity of the catalysts was evaluated by water decomposition to produce H_2_ under simulated sunlight and visible light using triethanolamine (TEOA) as a sacrificial agent. A quantity of 0.1 g of the sample was added to 100 mL of 20 wt.% TEOA solution and stirred continuously at 279 K. Then, the appropriate amount of H_2_PtCl_6_ was added and photo-deposited to ensure the photocatalysts were loaded with 3 wt.% Pt. Finally, a 300 W Xenon lamp with or without a filter (λ ≥ 420 nm) was used to irradiate the suspension. The generated H_2_ was detected with a gas chromatograph (Shimadzu, Kyoto, Japan, GC-2014C) equipped with a thermal conductivity detector (TCD). The apparent quantum yield (AQY) was determined by Equation (1):

(1) $$\mathrm{AQY}(\%)=\frac{{\mathrm{numbers}\;\mathrm{of}\;H}_{2}^{}\;\mathrm{molecules}\times 2}{\mathrm{numbers}\;\mathrm{of}\;\mathrm{photons}}\times 100$$

The number of photons (1.12 × 10^22^ photons·s^−1^) was measured by an irradiance meter (CELNP2000).

### 2.5. Density Functional Theory (DFT) Calculations

All calculations were performed with the DMol3 module of the Materials Studio 17.2. The generalized gradient approximation (GGA) with the Perdew–Burke–Ernzerhof (PBE) hybrid exchange-correlation function was utilized for geometry optimization, band structure, and density of state (DOS). In addition, the Van der Waals interaction was considered using Grimme’s DFT-D. The energy tolerance accuracy, maximum force, and displacement during the geometry optimization were set as 1.0 × 10^−5^ Ha, 0.002 Ha/Å, and 0.005 Å, respectively.

### 2.6. Calculation of Built-In Electric Field (BIEF)

The built-in electric field magnitude was calculated using Equation (2):

(2) $$F_{s}={\left(-2V_{s}\rho /\varepsilon \varepsilon_{0}\right)}^{1/2}$$

where *F_s_* is the internal electric field magnitude, *V_s_* is the surface potential, *ρ* is the surface charge density, *ε* is the low-frequency dielectric constant, and *ε*_0_ is the vacuum dielectric constant. The above equation reveals that the built-in electric field magnitude is mainly determined by the surface voltage and the surface charge density because *ε* and *ε*_0_ are two constants. The surface voltage can be characterized by the open circuit potential, while the surface charge density can be calculated using the zeta potential.

## 3. Results and Discussion

**Figure 1:**
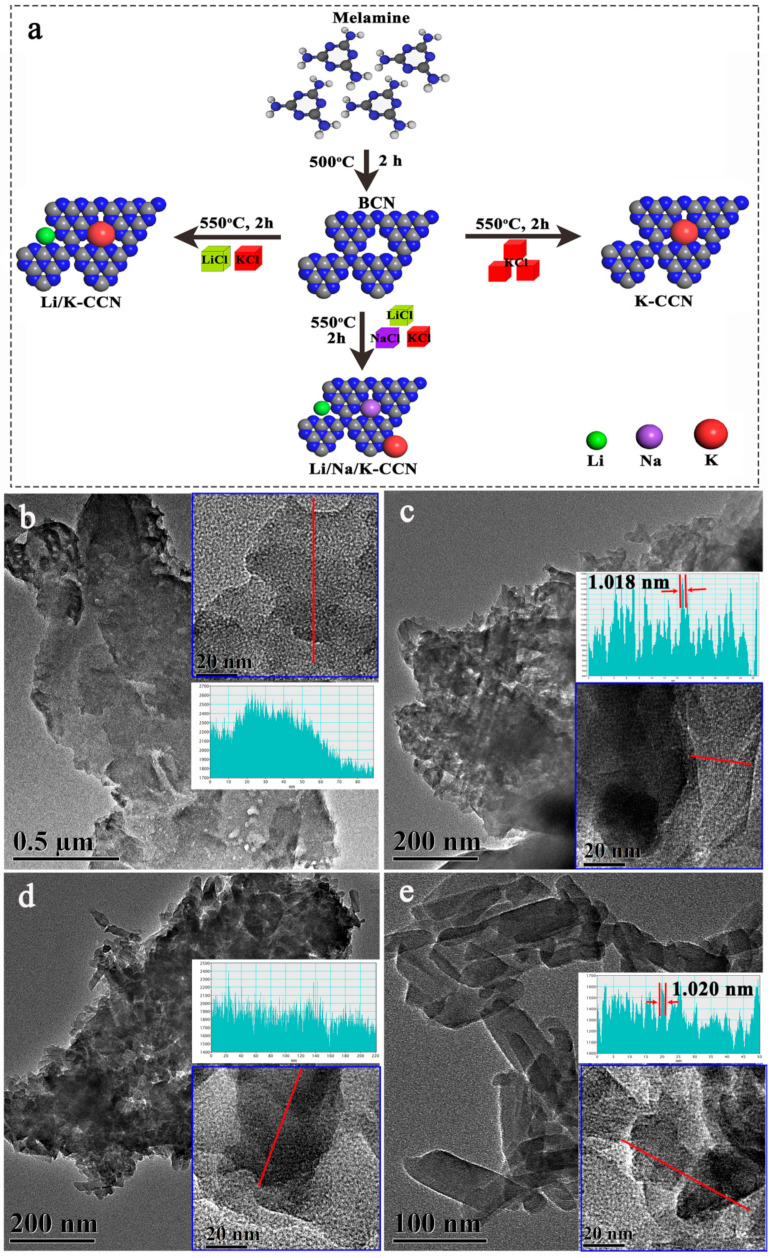
(**a**) Schematic illustration of the preparation of BCN, K-CCN, Li/K-CCN, and Li/Na/K-CCN by copolymerization of different molten salts. Transmission electron microscope (TEM) images and lattice profiles (insets) of (**b**) BCN, (**c**) K-CCN, (**d**) Li/K-CCN, (**e**) Li/Na/K-CCN.

The synthetic routes of BCN, K-CCN, Li/K-CCN, and Li/Na/K-CCN are depicted in Figure 1a. Firstly, the calcination of the melamine was adopted to prepare BCN, which was ground with different types of salts. The obtained homogeneous mixture was subsequently thermally treated at 550 °C to produce K-CCN, Li/K-CCN, and Li/Na/K-CCN samples. It is mentioned that solid LiCl, NaCl, and KCl possess melting points of 605, 801, and 770 °C, respectively, which are higher than the setting temperature (550 °C). The molten salts as solid templates can provide inter-crystal confined space to induce the structural rearrangement of BCN, thus enhancing the crystallinity of as-prepared BCN [22]. The detailed crystalline structures of four samples were investigated by TEM (Figure S1). As shown in Figure 1b, micrometer-scale layers with large amorphous aggregates were observed in BCN. The K-CCN sample possesses sheet-like features stacked in several layers (Figure 1c). The lattice fringe with a lattice spacing of 1.018 nm is labeled as the (020) facet of CCN, as seen from the high-magnification TEM inset and the lattice profile in Figure 1c, which validates the high crystallinity of K-CCN. As shown in Figure 1d, Li/K-CCN is an amorphous and micro-scale sheet, while Li/Na/K-CCN presents elongated sheets with a width of ~50 nm and length of ~100 nm. (Figure 1e). It can be observed from the inset TEM and lattice profile of Li/Na/K-CCN that a lattice fringe of 1.020 nm is assigned to the (020) facet of CCN. Compared with K-CCN, Li/Na/K-CCN exhibits incomplete and disorderly lattice stripes, indicating its significantly worse crystallinity compared to K-CCN.

**Figure 2:**
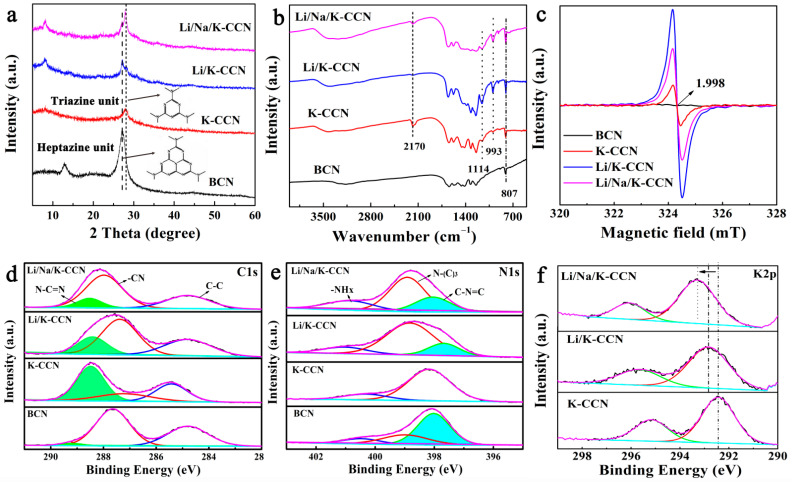
(**a**) X-ray diffraction (XRD) patterns, (**b**) Fourier transform infrared (FT-IR) spectra, (**c**) electron paramagnetic resonance (EPR) spectra, and X-ray photoelectron spectroscopy (XPS) of (**d**) C 1s, (**e**) N 1s, (**f**) K 2p of BCN, K-CCN, Li/K-CCN, and Li/Na/K-CCN.

**Figure 3:**
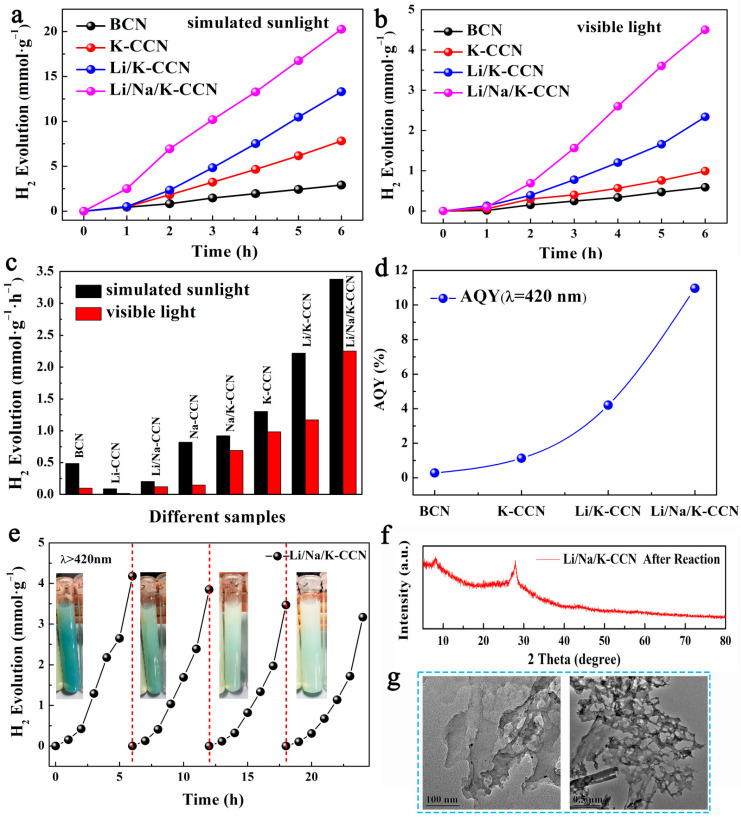
Time courses of photocatalytic hydrogen evolution under (**a**) simulated sunlight and (**b**) visible light of BCN, K-CCN, Li/K-CCN, and Li/Na/K-CCN, (**c**) the comparison chart of hydrogen evolution rate for different samples, (**d**) the apparent quantum yield (AQY) of BCN, K-CCN, Li/K-CCN, and Li/Na/K-CCN, (**e**) four cycling HER tests of Li/Na/K-CCN under visible-light irradiation, (**f**) XRD and (**g**) TEM images after four cycling HER tests of Li/Na/K-CCN.

As revealed in Figure 2a, two characteristic diffraction peaks at 27.2° and 13.8° are observed in the XRD pattern of BCN, indexed to (002) and (100) facets of g-C_3_N_4_, which represent the in-plane stacking of the C_3_N_4_ layer and the repeated heptazine units, respectively [34]. Compared with BCN, K-CCN shows the peak shift of the (002) facet from 27.2° to 28.1°, indicating the narrowed interlayer distance due to the introduction of K^+^ ions in BCN [35]. The peak at 13.8° is absent in K-CCN, and a new peak appears at 8.1°, corresponding to the in-plane repeating triazine units with a lattice stripe of about 1.103 nm. The average grain size of Li/Na/K-CCN nanocrystals calculated by Scherrer broadening is about 0.30 nm, which is larger than that of BCN (0.12 nm), and the crystallinity calculated by XRD is 87%, which is larger than that of BCN (77%). The above results further prove the high crystallinity of Li/Na/K-CCN. For the diffraction patterns of Li/K-CCN and Li/Na/K-CCN in Figure 2a, four characteristic peaks at 8.1°, 13.8°, 27.2°, and 28.1° are observed, which indicates that both heptazine and triazine units exist in Li/K-CCN and Li/Na/K-CCN, and heptazine-based units dominate in Li/K-CCN, while triazine-based units are in the majority in Li/Na/K-CCN. In the FTIR spectra of BCN, K-CCN, Li/K-CCN, and Li/Na/K-CCN (Figure 3a), the peak at 807 cm^−1^ represents the bending vibrations of the heptazine or triazine rings, while the characteristic peaks in the range of 1200–1700 cm^−1^ are attributed to the characteristic stretching and bending vibrations of the conjugated heterocycle in g-C_3_N_4_ [36]. New peaks appearing at 993 cm^−1^ and 1114 cm^−1^ in K-CCN, Li/K-CCN, and Li/Na/K-CCN are due to the intercalation of K^+^ and the bending vibration of N-H, respectively. In addition, new bands at 2170 cm^−1^ for K-CCN, Li/K-CCN, and Li/Na/K-CCN are attributed to the stretching vibrations of the terminal cyano group (C≡N), which originated from the decomposition/polymerization of the heptazine C_3_N_4_ [37]. Notably, the cyano groups as strong electron-withdrawing groups would produce a BIEF for promoting the exciton dissociation [38].

The structural defects of the as-obtained four samples were investigated using an EPR spectrometer. As seen in Figure 2c, the symmetrical peaks at g = 1.998 are assigned to the unpaired electrons produced by the partial defects on aromatic rings in the g-C_3_N_4_ framework [39]. Obviously, the intensity of the EPR signal follows the order of Li/K-CCN > Li/Na/K-CCN > K-CCN > BCN, which indicates that the addition of salt ions increases the defects in the C_3_N_4_ skeleton. Based on the XRD patterns in Figure 2a, it can be seen that the differences in defects are attributed to their different crystal structures. Li ions with a small size (59 pm) can enter the interior of the g-C_3_N_4_ skeleton, thus resulting in the destruction of triazine-based units and the generation of enriched defects in Li/K-CCN, whereas the intercalation of K ions can promote the conversion of the C_3_N_4_ structure from heptazine-based units to triazine-based units, and K ions mainly aggregate on the surface due to larger sizes (137 pm), causing the limited defects concentrated on the surface. Interestingly, the size of Na^+^ (99 pm) lies between that of Li^+^ and K^+^, and they can be intercalated into the location between the surface and interior, resulting in the retention of most triazine units and moderate defects in Li/Na/K-CCN. As illustrated in Table 1, the molar proportion of Li/Na/K in bulk is 1:0.27:0.75 from the ICP-MS results, which changes to 1:0.73:3.24 on the surface, as seen from the XPS results. This further confirms that K is mainly distributed on the surface of the g-C_3_N_4_ framework, and Na is distributed more inward, while Li can enter the interior of the g-C_3_N_4_ framework. As shown in Figure 2d, the C 1s spectrum can be fitted into three peaks located at 288.2, 286.7, and 284.8 eV, which originate from the aromatic N-C=N, C≡N, and graphitic C-C, respectively [37]. Significantly, the proportion of N-C=N for the triazine unit in C 1s XPS spectra changes from trace (BCN) to primary (K-CCN) and then to secondary (Li/K-CCN and Li/Na/K-CCN). In N 1s XPS spectra (Figure 2e), the two fitted peaks at 400.6 and 399.2 eV are assigned to -NH_x_ and N-(C)_3_, respectively [40]. In addition, the peak at 398.1 eV corresponds to C-N=C in the heptazine unit [41], which shows the domination in BCN, absence in K-CCN, and incidental presence in Li/K-CCN and Li/Na/K-CCN. As displayed in Figure 2f, the two peaks at 295.2 and 292.4 eV can be indexed to K 2p of K^+^, which moves towards high binding energy with the addition of Li and Na ions. It is indicated that the addition of combined salts induces the formation of BIEF, thus resulting in the attraction between K^+^ and adjacent layers [35]. The above results prove that introducing different salt ions has various effects on the g-C_3_N_4_ structure. K ions mainly induce the reconstruction of the g-C_3_N_4_ skeleton from the heptazine unit to the triazine unit, thereby improving the crystallinity of samples, as seen from the XRD patterns in Figure 2a. The intercalation of Li and Na ions causes structural defects (Figure 2c), as well as partial retention of the heptazine unit, resulting in the simultaneous presence of heptazine to triazine units and the formation of BIEF in Li/K-CCN and Li/Na/K-CCN.

The photocatalytic performance of the four as-obtained catalysts was explored by H_2_ evolution reaction (HER). As seen in Figure 3a,b, the four samples present linear H_2_ evolution for six hours under simulated sunlight and visible-light irradiation. The HER rates of the samples doped with different molten salts are displayed in Figure 3c. The HER rate of the Li/Na/K-CCN sample is 3.38 mmol·g^−1^·h^−1^ under simulated sunlight, which is 1.5-fold, 2.6-fold, and 7-fold of Li/K-CCN (2.22 mmol·g^−1^·h^−1^), K-CCN (1.30 mmol·g^−1^·h^−1^), and BCN (0.48 mmol·g^−1^·h^−1^), respectively. Under visible-light irradiation, the HER rate of Li/Na/K-CCN (2.25 mmol·g^−1^·h^−1^) is much higher than that of Li/K-CCN (1.17 mmol·g^−1^·h^−1^), K-CCN (0.98 mmol·g^−1^·h^−1^), and BCN (0.10 mmol·g^−1^·h^−1^). In addition, the photocatalytic hydrogen production rate of the prepared Li/Na/K-CCN sample in this paper is superior to that of most other photocatalysts published in the literature, as shown in Table 2. As shown in Figure 3c, the addition of K ions to CCN significantly increased HER, while the addition of Na^+^ did not significantly improve the rate, and the insertion of Li^+^ actually reduced the HER rate. Meanwhile, the complete photocatalytic hydrogen production performance of molten salt CCN (Figure S2) also supported the above conclusion. Surprisingly, when Li^+^, Na^+^, and K^+^ are mixed in CCN, the HER rate is significantly improved. The AQY values at λ = 420 nm are presented in Figure 3d, which indicates the AQY of Li/Na/K-CCN at λ = 420 nm is calculated to be 10.97%, which is 40.6 times higher than that of BCN (0.27%). Additionally, as shown in Figure 3e, Li/Na/K-CCN demonstrates a 23.8% reduction after four cycles within 24 h, which is related to the continuous detachment of K ions from the catalyst during the HER process, as can be seen from the gradual fading of the blue color in the inset photos. Furthermore, the XRD pattern (Figure 3f) and TEM images (Figure 3g) of the recycled Li/Na/K-CCN show that the crystallinity and morphology of the Li/Na/K-CCN sample are well preserved, and the composition is still composed of heptazine and triazine units, indicating the excellent structural stability of Li/Na/K-CCN. In addition, compared with triethanolamine, the hydrogen production activity of Li/Na/K-CCN decreases significantly when methanol is used as the sacrificial agent (Figure S3), which is related to the lone pair electrons on triethanolamine nitrogen atoms.

**Figure 4:**
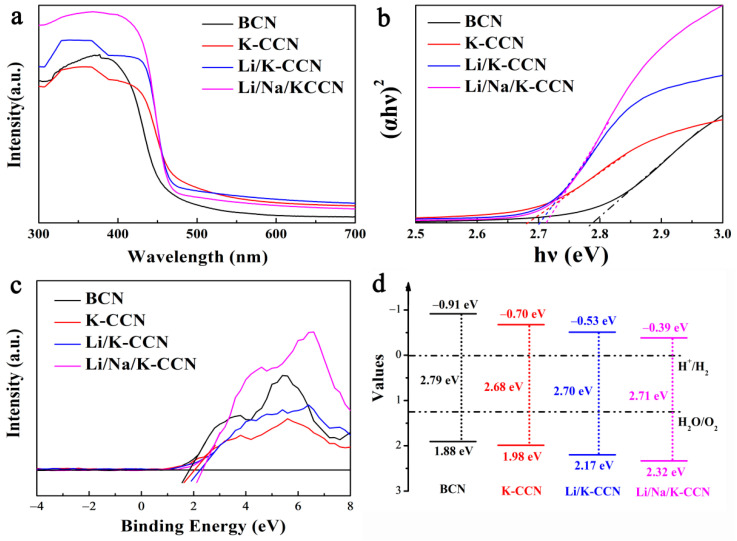
(**a**) UV-visible absorption spectra, (**b**) corresponding band gap energies, (**c**) VB-XPS plots, and (**d**) simplified band structures of BCN, K-CCN, Li/K-CCN, and Li/Na/K-CCN.

As seen in Figure 4a, the salt-ion-doped CCN samples (K-CCN, Li/K-CCN, and Li/Na/K-CCN) exhibit a wider and more intense optical absorption than BCN, which is attributed to the improved crystallinity and extended π-conjugated structure [44]. Accordingly, the bandgaps of BCN, K-CCN, Li/K-CCN, and Li/Na/K-CCN are determined to be 2.79, 2.68, 2.70, and 2.81 eV, respectively (Figure 4b). The valence band (VB) positions of the four samples were estimated from the XPS valence spectra. As demonstrated in Figure 4c, the VB level of Li/Na/K-CCN increases to 2.32 eV as compared to BCN (1.88 eV). Based on the bandgaps, the conduction bands (CB) of BCN, K-CCN, Li/K-CCN, and Li/Na/K-CCN are calculated to be −0.91, −0.70, −0.53, and −0.39 eV, respectively. As illustrated in Figure 4d, the obvious up-shifts of CB and VB are observed from BCN to K-CCN and then to Li/Na/K-CCN, which would reduce the reduction ability of photo-induced electrons. However, from the result of the photocatalytic HER in Figure 3, the H_2_ production rate of Li/Na/K-CCN is much superior to others, which demonstrates that the heterojunction construction between heptazine and triazine units overcomes the reduction in redox potential and plays a dominant role in the photocatalytic HER. Based on the matching energy level of heptazine-based BCN and triazine-based K-CCN in Figure 4d, a type-II heterojunction would be constructed in Li/K-CCN and Li/Na/K-CCN, resulting in further improvement in HER performance.

**Figure 5:**
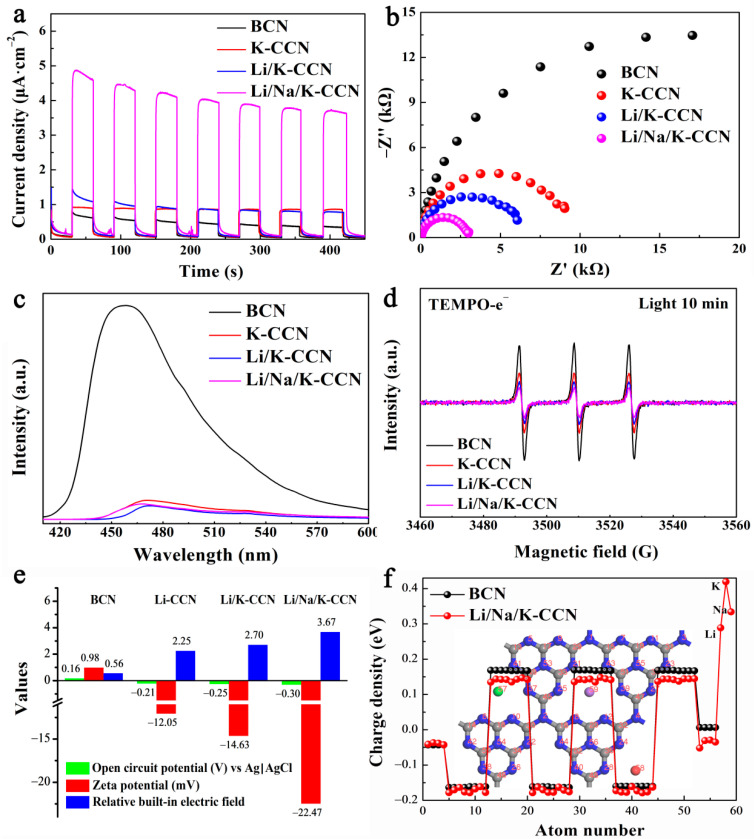
(**a**) The transient photocurrent responses, (**b**) EIS Nyquist plots, (**c**) steady state photoluminescence spectra, (**d**) in situ electron spin resonance (ESR) signals labeled by TEMPO for electrons, (**e**) the calculated built-in electric field of BCN, K-CCN, Li/K-CCN, and Li/Na/K-CCN, and (**f**) the optimized charge density by DFT of BCN and Li/Na/K-CCN.

The photo-current curves of BCN, K-CCN, Li/K-CCN, and Li/Na/K-CCN are revealed in Figure 5a. Among the four samples, Li/Na/K-CCN exhibits the optimal photocurrent density, owing to the rapid separation of photo-generated carriers and the increased charge migration in the type-II Li/Na/K-CCN heterojunction. Furthermore, as revealed in Figure 5b, the semi-circular radius of the Nyquist plots of four samples indicated the order of BCN > K-CCN > Li/K-CCN > Li/Na/K-CCN. It is demonstrated that the high crystallinity of g-C_3_N_4_ could reduce the impedance of catalysts; in particular, the formation of heterojunctions would further enhance the conductivity of samples through built-in electric fields. As seen in Figure 5c, BCN exhibits a wide and strong PL emission peak centered at 457 nm, ascribed to the rapid recombination of photo-induced charge carriers [43]. Contrarily, the three CCN samples present a similar low emission intensity, demonstrating the discernible improved separation of the radiative carriers. TEMPO spin-labeled ESR was investigated to further demonstrate the efficient photo-generated charge separation of Li/Na/K-CCN. As seen in Figure 5d, the weakened ESR signal of Li/Na/K-CCN is attributed to the electrons captured by TEMPO, which indicates that more carriers are induced in the Li/Na/K-CCN sample [42]. Meanwhile, it can be clearly seen in Figure S4 that, with the increase in the light radiation time, a wider ESR spectral peak is formed, which indicates that the production of electrons depends on the irradiation of light. Based on the calculation method of BIEF reported by Zhang et al. [45], the intensity of BIEF of the four samples was calculated from the open circuit potential (vs. NHE) and zeta potential, as illustrated in Figure 5e. Briefly, the relative BIEF values for BCN, K-CCN, Li/K-CCN, and Li/Na/K-CCN are 0.56, 2.25, 2.70, and 3.67, respectively. Moreover, the existence of BIEF was simulated by the DFT calculations. The atomic charge densities of BCN and Li/Na/K-CCN are exhibited in Figure 5f, and the corresponding atomic numbers in Li/Na/K-CCN are presented in the background of Figure 5f, which implies the charge density in Li/Na/K-CCN undergoes a polarized distribution compared with that of BCN. The results in Figure 5 demonstrate that Li/Na/K-CCN has an enhanced BIEF via the formation of type-II heterojunctions, which greatly boosts the photocatalytic performance by promoting photo-induced charge generation, separation, and migration.

**Figure 6:**
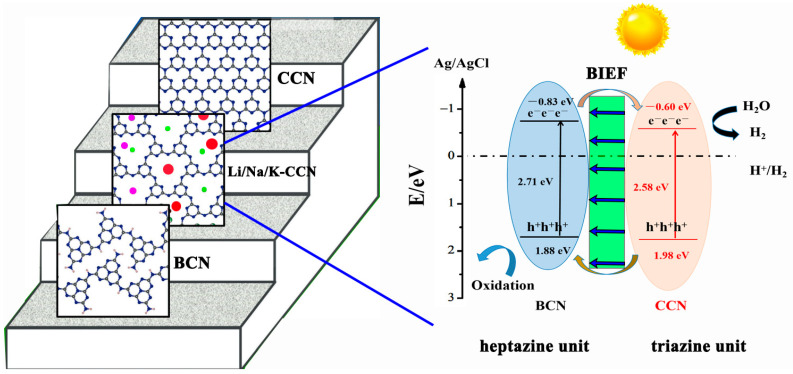
Possible routes for the separation of photo-generated electron–hole pairs and their transfer in the triazine/heptazine-based Li/Na/K-CCN heterojunction.

According to the above results, the heptazine-based unit is dominant in the BCN sample, which processes a greater negative energy level (−0.83~1.88 eV) and higher photocatalytic reduction ability, whereas the photo-generated charge carriers will be easier to recombine. Regarding the high-crystalline K-CCN sample, triazine-based units are in the majority, resulting in the up-shift of the energy level (−0.60~1.98 eV) and fast charge transfer. Nevertheless, heptazine and triazine units co-exist in Li/K-CCN and Li/Na/K-CCN samples. Therefore, a proposed mechanism for the photocatalytic HER process of the Li/Na/K-CCN sample is illustrated in Figure 6. The heptazine-based BCN and triazine-based CCN in the Li/Na/K-CCN sample have staggered band structures. Accordingly, these two types of g-C_3_N_4_ unit will spontaneously form type-II heterojunctions with a BIEF, thus enhancing the migration of photo-induced charges, and further improving the photocatalytic activity.

## 4. Conclusions

In summary, K-CCN, Li/K-CCN, and Li/Na/K-CCN catalysts were obtained using pre-prepared BCN as the precursor in the molten salt approach. The structures of the prepared Li/K-CCN and Li/Na/K-CCN samples were optimized and co-existed with two isotypes (heptazine and triazine), which constructed a BIEF and formed a type-II heterojunction. As a result, the prepared Li/Na/K-CCN sample had a HER rate of 3.38 mmol·g^−1^·h^−1^ under simulated sunlight irradiation and 2.25 mmol·g^−1^·h^−1^ under visible-light irradiation, which are both significantly higher than those of BCN and other CCN catalysts. The boosted photocatalytic activity of the Li/Na/K-CCN sample is mainly ascribed to the construction of the type-II heterojunction and the formation of BIEF in triazine/heptazine-based Li/Na/K-CCN, which significantly enhances the photo-induced charge generation, separation, and migration. This study may enable facile synthesis and further optimization for improving the charge mobility of crystalline PCN via the construction of heterojunctions.

## Figures and Tables

**Table 1:** Bulk and surface Li, Na, and K contents in Li/Na/K-CCN obtained from ICP-MS and XPS.

Technique	Elemental Contents (at.%)		
	Li	Na	K
XPS	1.21	0.88	3.92
ICP-MS	0.1843	0.0499	0.1377

**Table 2:** Comparison of photocatalytic H_2_ production performance of different catalysts.

Photocatalyst	Amt. (mg)	Experimental Conditions	Light Source	H_2_ Evolution (μmol·g^−1^·h^−1^)	Reference
MC-CN	100	10% TEOA solution, 3% Pt	300 W Xe lamp	1125	[21]
tri-/tri-s-tri-C_3_N_4_-90	50	10% TEOA solution, 3% Pt	300 W Xe lamp (>420 nm)	36	[30]
P3/CN	50	17% TEOA solution	300 W Xe lamp	13,000	[16]
MS-550	100	10% TEOA solution, 3% Pt	300 W Xe lamp (>420 nm)	661	[35]
PbTiO_3_-TiO_2_	30	20% methanol solution	500 W high-pressure mercury lamp (<420 nm)	21,017	[3]
CN2	10	10% TEOA solution, 3% Pt	300 W Xe lamp (>420 nm)	1271	[13]
1.8PCN	20	18% lactic acid aqueous solution	300 W Xe lamp (≥400 nm)	571	[28]
CCNNSs	50	10% methanol solution, 3% Pt	monochromatic LED lamps (>420 nm)	1060	[33]
CN-SPO	10	20% TEOA solution, 3% Pt	300 W Xe lamp (>420 nm)	2479	[42]
CN_16_	20	15% TEOA solution, 0.5% Pt	300 W Xe lamp (>420 nm)	2025	[19]
CNSC	10	12% methanol solution, 3% Pt	300 W Xe lamp (>400 nm)	3960	[43]
Li/Na/K-CCN	100	20% TEOA solution, 3% Pt	300 W Xe lamp (>420 nm)	2250	This work

## Data Availability

Not applicable.

## Supplementary Materials

The following supporting information can be downloaded at: https://www.mdpi.com/article/10.3390/nano13162300/s1. Figure S1: TEM images of (a) BCN, (b) K-CCN, (c) Li/K-CCN, and (d) Li/Na/K-CCN. Figure S2: Photocatalytic hydrogen production activity of crystalline carbon nitride polymerized with different molten salts under (a) simulated sunlight and (b) visible light. Figure S3: Photocatalytic H_2_ production of the Li/Na/K-CCN sample using methanol and triethanolamine as sacrificial agents under simulated sunlight. Figure S4: Electron spin resonance (ESR) spectra of electrons captured by TEMPO at different times under visible-light irradiation.
