# Supplementary material for: Construction of Type-II Heterojunctions in Crystalline Carbon Nitride for Efficient Photocatalytic H2 Evolution
Source: Nanomaterials (Basel). 2023 Aug 10;13(16):2300. doi: 10.3390/nano13162300 (PMC10459030; doi:10.3390/nano13162300)
Supplement: Supplementary file 1 [file nanomaterials-13-02300-s001.zip › nanomaterials-2533652-supplementary.pdf]

Supplementary Material

Construction of Type-II Heterojunction in Crystalline Carbon Nitride for Efficient Photocatalytic H_2 Evolution

Jingyu Zhang, Zhongliang Li, Jialong Li, Yalin He, Haojie Tong, Shuang Li, Zhanli Chai*and Kun Lan*

Inner Mongolia Key Laboratory of Chemistry and Physics of Rare Earth Materials, College of Chemistry and Chemical Engineering, College of Energy Materials and Chemistry, Inner Mongolia University, Hohhot 010021, China

* Correspondence: chai_zl@hotmail.com; k_lan@imu.edu.cn

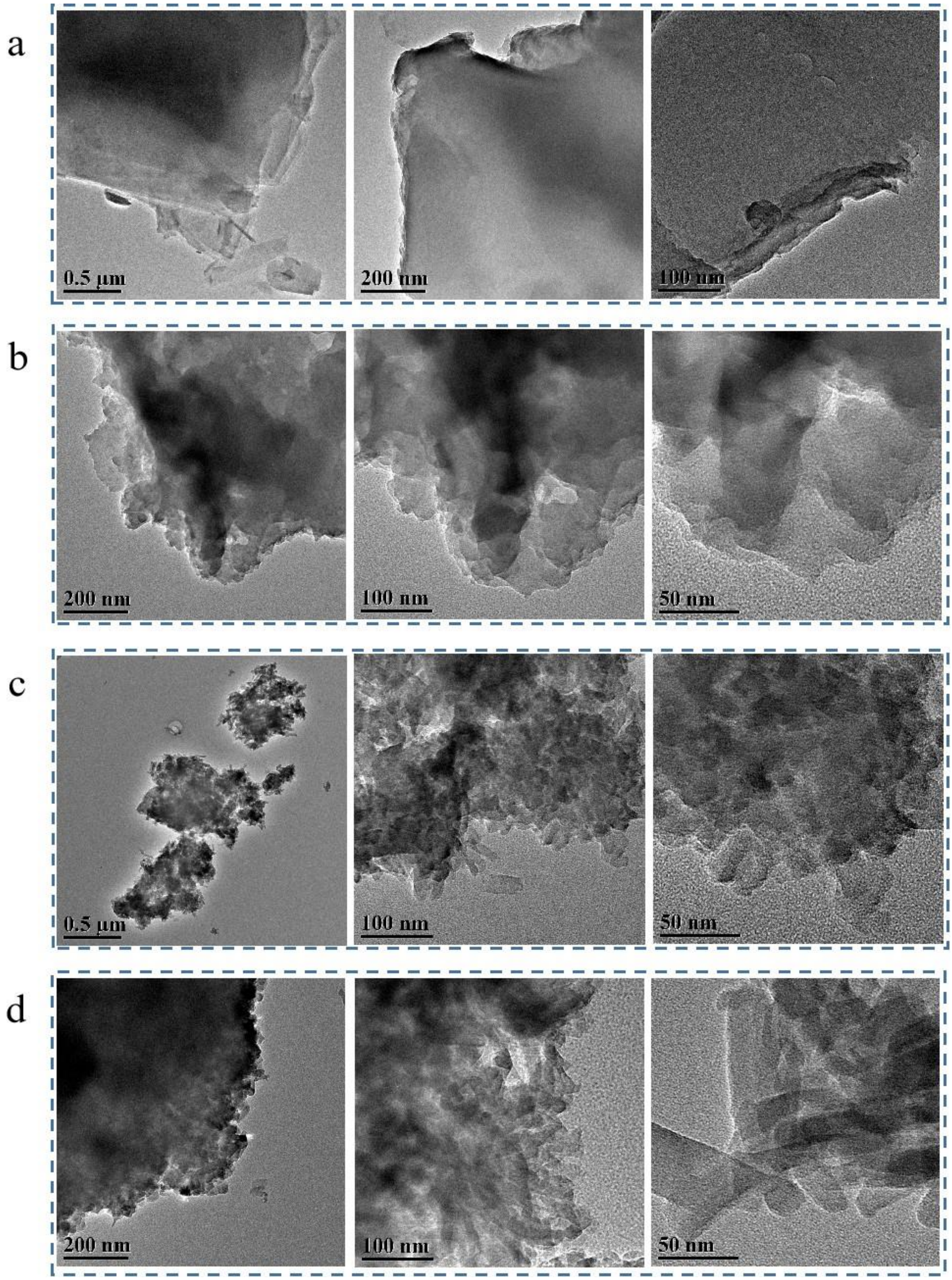

Figure S1. TEM images of (a) BCN, (b) K-CCN, (c) Li/K-CCN, and (d) Li/Na/K-CCN.

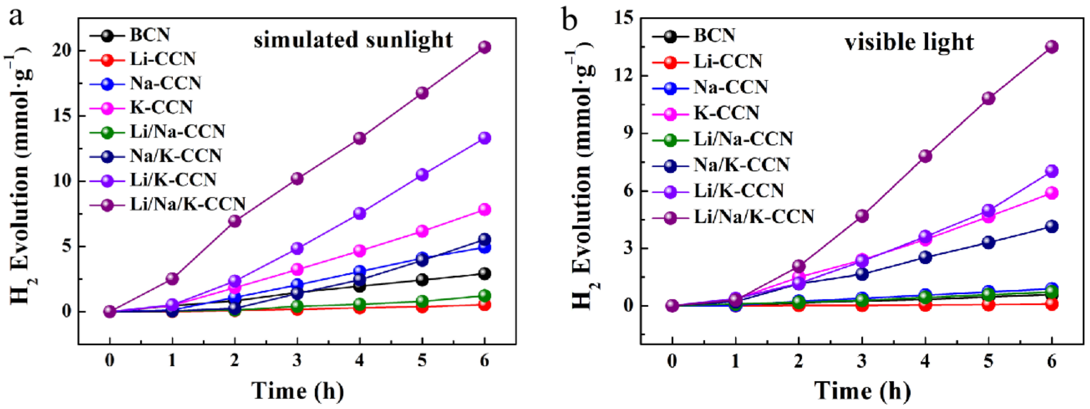

Figure S2. Photocatalytic hydrogen production activity of crystalline carbon nitride polymerized with different molten salts under (a) simulated sunlight and (b) visible light.

Li-CCN and Na-CCN are 13.2 g anhydrous LiCl and NaCl, respectively, copolymerized with 2.0 g as-prepared BCN, and calcined at 550 °C for 2 h. Li/Na-CCN and Na/K-CCN were obtained by copolymerization of 20.0 g double molten salt (LiCl: NaCl=1:1) and (NaCl: KCl=1:1) with 2.0 g as-prepared BCN and calcined at 550 °C for 2 h.

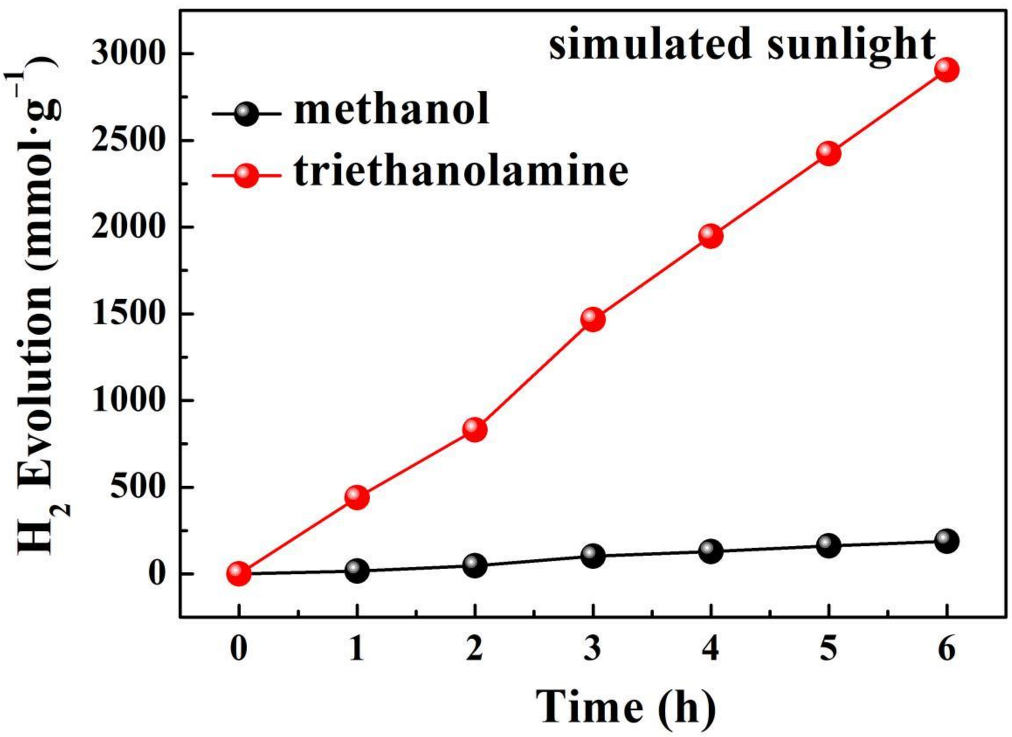

Figure S3. Photocatalytic H_2 production of the Li/Na/K-CCN sample using methanol and triethanolamine as sacrificial agents under simulated sunlight.

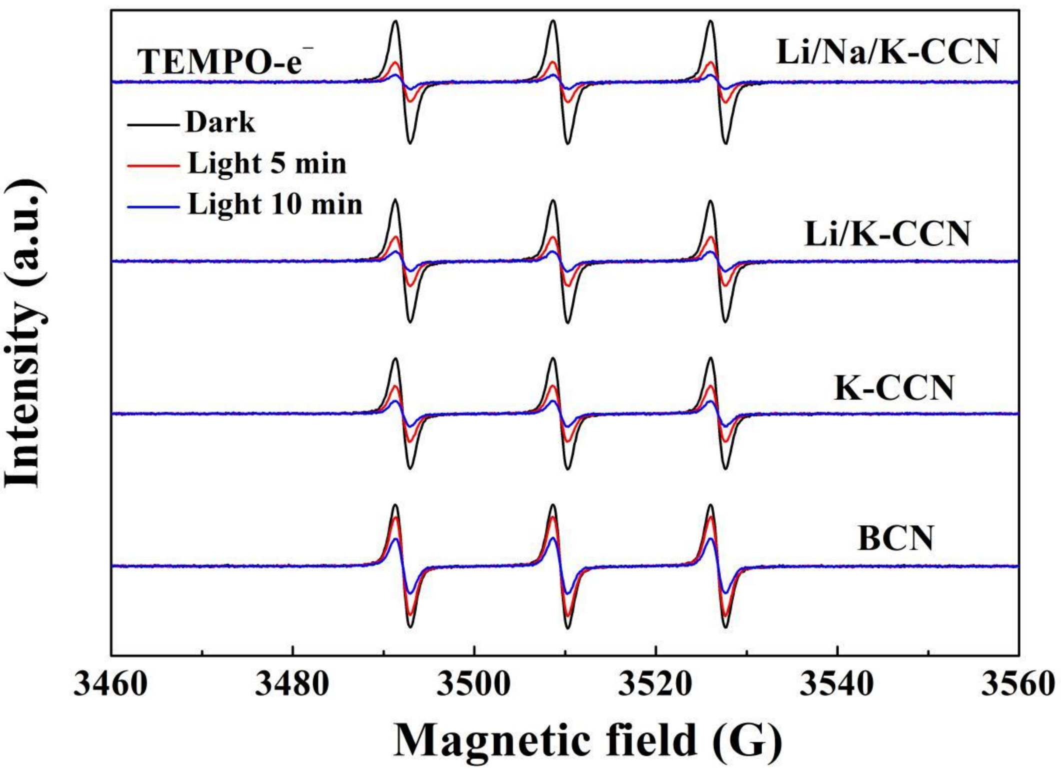

Figure S4. Electron spin resonance (ESR) spectra of electrons captured by TEMPO at different times under visible light irradiation.
